# Tumor infiltrating B lymphocytes (TIBs) associate with poor clinical outcomes, unfavorable therapeutic benefit and immunosuppressive context in metastatic clear cell renal cell carcinoma (mccRCC) patients treated with anti-PD-1 antibody plus Axitinib

**DOI:** 10.1007/s00432-024-05803-5

**Published:** 2024-05-19

**Authors:** Zhiyuan Lin, Shuxiu Xiao, Yu Qi, Jianming Guo, Lili Lu

**Affiliations:** 1 Department of Urology, Zhongshan Hospital, Fudan University, Shanghai, China; 2 Clinical Center for Biotherapy, Zhongshan Hospital, Fudan University, Shanghai, China

**Keywords:** Tumor infiltrating B lymphocyte, Clear cell renal cell carcinoma, PD-1, Axitinib, Survival

## Abstract

**Purpose:**

Immune checkpoint inhibitors (ICIs) plus tyrosine kinase inhibitors (TKIs) has become first-line therapy for metastatic renal cell carcinoma patients. This study aims to investigate the effect of tumor infiltrating B lymphocytes (TIBs) on the combination therapy.

**Methods:**

The retrospective analysis was conducted on the clinical records of 115 metastatic clear cell renal cell carcinoma (mccRCC) patients treated with anti-PD-1 antibody plus Axitinib between March 2020 and June 2023. Observation target: objective response rate (ORR), and overall survival (OS), progression-free survival (PFS) and immune profile.

**Results:**

Patients with high TIBs portended lower ORR of the combination therapy (p = 0.033). TIBs was an independent predictor for poorer OS (p = 0.013) and PFS (p = 0.021) in mccRCC patients with combination treatment. TIBs infiltration was associated with more CD4^+^T (p < 0.001), CD8^+^T (p < 0.001), M2 macrophages (p = 0.020) and regulatory T cells (Tregs) (p = 0.004). In TIBs high patients, the percentages of PD-1, CTLA-4 and TIM-3 positive rate were significantly increased in CD4^+^T (p = 0.038, 0.029 and 0.002 respectively) and CD8^+^T cells (p = 0.006, 0.026 and < 0.001 respectively).

**Conclusions:**

Our study revealed TIBs infiltration predicted adverse outcomes in mccRCC patients treated with anti-PD-1 antibody plus Axitinib. As a corollary, TIBs positively associated with M_2_ macrophages and Tregs, leading to subsequent multiple immune checkpoints related exhaustion of T cells. Thus, only PD-1 blockade are inadequate to reverse T cells exhaustion effectively in high TIBs mccRCC patients.

**Supplementary Information:**

The online version contains supplementary material available at 10.1007/s00432-024-05803-5.

## Introduction

Renal cancer accounts for 5% of all tumors and its incidence continued to increase annually in both gender (Siegel et al. 2023). Renal cell carcinoma (RCC) is the most common type of renal cancer. Since high proportion of asymptomatic cases, 25–30% RCC patients had metastases at the time of initial diagnosis (Usher-Smith et al. 2020). The prognosis significantly deteriorated if the disease turn into metastatic renal cell carcinoma (mRCC).

Tyrosine kinase inhibitors (TKIs) were used as first-line treatment for mRCC, which had significantly improved the prognosis of these patients (Motzer et al. 2007). Recent years, clinical trials like CLEAR, CheckMate 9ER and KEYNOTE-426 study has revolutionized the therapeutic strategy (Choueiri et al. 2023; Motzer et al. 2022; Plimack et al. 2023). Immune checkpoint inhibitors (ICIs) plus TKIs has become first-line therapy for mRCC patients. The objective response rate (ORR) had reached over 50% in these studies mentioned above. Tumor mutation burden, PD-L1 expression and microsatellite instability (MSI) had been approved to be predictors for ICIs therapy (Havel et al. 2019). However, nearly all previous studies focused on the role of indicators in TKI or ICI monotherapy respectively. Little research explore predictors of the “ICI + TKI” combination therapy.

The biological and clinical relevance of T lymphocytes in anti-tumor immune response and immunotherapy has been well characterized. As another subtype of adaptive immune cells, the function of B lymphocytes is often overlooked. For a long time, B cells are recognized as the main effector cells of humoral immunity and suppress cellular immunity. Emerging data have shown that B cells play a complex role in tumor immunity. On one hand, regulatory B cells (Bregs) subsets express immune inhibitory cytokines such as IL-10, TGF-β and IL-35 (Kessel et al. 2012; Pylayeva-Gupta et al. 2016; M. Zhou et al. 2016a, b). Additionally, Bregs express a variety of suppressive ligands including PD-L1, PD-1 and LAP-TGF-β, which can suppress immune effector cells response (Lee-Chang et al. 2019; Xiao et al. 2016; Zhang et al. 2016). On the other hand, Intratumoral B cells may drive antibody-dependent cellular cytotoxicity (ADCC) and enhance antigen presentation by secreting IgG antibodies (Carmi et al. 2015). B cell can also work as anti-tumor cytokine producer and even direct tumor killer to limit tumor progression (Shi et al. 2013; Tao et al. 2015).

A recent study showed that high CD20^+^ B cells infiltration identifies a poor prognosis subset of RCC patients (Sjoberg et al. 2018). Whereas, another researches suggested TIBs predicted longer overall survival (OS), progression-free survival (PFS) and better therapeutic response in TKI-treated mRCC patients (Lin et al. 2018). In addition, the effect of TIBs on immunotherapy response is also contradictory. High B cells infiltration improved soft-tissue sarcomas, melanoma and RCC patients survival and predicted a high response rate to anti-PD-1 therapy (Helmink et al. 2020; Petitprez et al. 2020). However, another study showed B cell depletion or absence is disassociated with response to anti-PD-1 inhibitors in melanoma (Damsky et al. 2019). Therefore, the role of B cells in ICI therapy remains to be revealed.

Clear cell renal cell carcinoma (ccRCC) is the most common pathological subtype of RCC, accounting for 70–80% of all RCC cases. In this study, we aim to assess the prognostic value of TIBs in metastatic ccRCC (mccRCC) patients treated with anti-PD-1 antibody combined Axitinib.

## Patients and methods

### Patient population

**Fig. 1:**
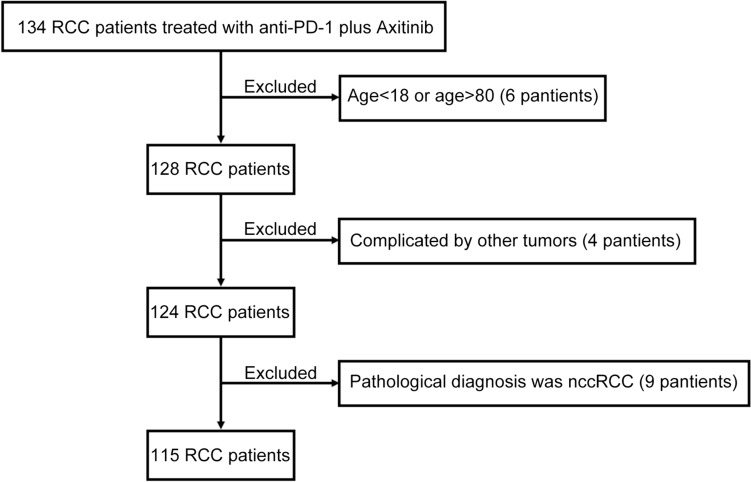
Flow diagram of the cohort involved in this study

RCC patients treated with anti-PD-1 antibody plus Axitinib combination therapy was retrospectively screened in the Department of Urology, Zhongshan Hospital, Fudan University. 134 RCC patients who underwent anti-PD-1 antibody (Pembrolizumab or Tislelizumab) plus Axitinib between March 2020 and June 2023 were identified as potentially relevant. Six pantients were excluded because of inapposite age (< 18 or > 80). Four pantients were excluded because of complicated by other tumors. Then, nine patients were excluded because the pathological diagnosis was non-clear cell renal cell carcinoma (nccRCC). Finally, 115 ccRCC patients were ultimately included in the analysis (Fig. 1). The median follow-up was 18 months (2–42 months) and the major characteristics were listed in Table 1. All enrolled patients had received primary tumor resection (radical or cytoreductive nephrectomy). Metastasectomy was also performed in 35 suitable patients.

**Table 1:** Clinical characteristics of patients according to TIBs number

Characteristics	Patients		TIBs		
	n	%	Low	High	p
All patients	115	100	80	35	
Age*					0.792
< 65	57	49.6	39	18	
≥ 65	58	50.4	41	17	
Gender					0.679
Female	46	40	33	13	
Male	69	60	47	22	
Initial TNM stage**					0.176
I–II	42	36.5	26	16	
III–IV	73	63.5	54	19	
ISUP grade **					0.186
1–2	60	52.2	45	15	
3–4	55	47.8	35	20	
Number of metastatic organs***					0.532
1	74	64.3	50	24	
≥ 2	41	35.7	30	11	
Metastasectomy					0.878
Yes	35	30.4	24	11	
No	80	69.6	56	24	
previous TKI usage***					0.408
Yes	46	40	34	12	
No	69	60	46	23	
IMDC risk stratification***					0.043
Favorable risk	36	31.3	22	14	
Intermediate risk	68	59.1	53	15	
Poor risk	11	9.6	5	6	
Anti-PD-1 antibody					0.562
Pembrolizumab	44	38.3	32	12	
Tislelizumab	71	61.7	48	23	

^*^Age at initiation of systemic therapy, split at median; **determined after primary tumor resection; ***determined at the anti-PD-1 antibody plus Axitinib combination therapy; p < 0.05 was regarded as statistically significant

Kidney Renal Clear Cell Carcinoma (KIRC) cohort from The Cancer Genome Atlas (TCGA) was downloaded from TCGA database (https://cancergenome.nih.gov/). Absolute proportion of immune cells in TCGA Cohort was obtained by CIBERSORT calculation (Chen et al. 2018). Absolute proportion of TIBs was defined as the sum of absolute proportion of B cells naïve, B cells memory and plasma cells calculated by CIBERSORT.

### Immunohistochemistry (IHC) staining and evaluation

IHC staining were applied to identifiy specific cells. Tissue microarray slides were scanned on NanoZoomer-XR (Hamamatsu). Immune cell number was counted by two urologists (blinded to the clinical data) independently. The density of positive staining cells was calculated (cells/mm^2^) and used for grouping and analyzing. Details of IHC antibodies were shown in Table S1.

### Flow cytometry (FCM)

Fresh ccRCC tissues (at least 1 cm^3^ away from the tumor site) were minced and digested with collagenase IV to prepare single cell suspensions, and followed incubated by RBC lysis buffer (BD Biosciences). Then, single cells were stained with appropriate monoclonal antibodies for 30 min at 4 degree centigrade. After disposed by Fixation/Permeabilization Solution Kit (BD Biosciences) according to manufacture protocol. Cell suspensions were stained with fluorochrome-labeled antibodies and preserved with cell staining buffer. FCM was performed with a BD FACScelesta, and cells were analyzed using FlowJo software (Tree Star). Details of FCM antibodies were shown in Table S2.

### Statistical analyses

Baseline demographic, histological and clinical data were collected from medical records, electronic databases and follow-up information. The tumor histological type and stage according to the 2016 WHO criteria and the definitions of the 8th edition of the American Joint Committee on Cancer (AJCC) stage classification (Cornejo et al. 2020; Delahunt et al. 2019). Disease progression was identified via RECIST1.1 criteria (Eisenhauer et al. 2009). OS was defined as the time from combined therapy to death or the last observation. PFS was calculated from the date of combined therapy to disease progression, mortality or the last observation.

The cut point of TIBs density was determined at 5.0/mm^2^ using X-tile software version 3.4.7 (Robert Camp 2005) through minimum p value method based on the patients’ OS information (Camp et al. 2004). Statistical analyses were performed using SPSS 21.0 (SPSS Inc.) in this study. The association between cells infiltration and baseline characteristics were assessed by the chi-square test method. The Kaplan–Meier method and log rank test was applied to survival analyses. Univariate- and multivariate-Cox proportional hazard models were used to identify independent prognostic factors in sequence. Pearson’s correlation test was used to evaluate the associations between immune cells. 2-tailed p < 0.05 was considered as statistically significant.

## Results

### TIBs infiltration predicted lower ORR in combination therapy

**Fig. 2:**
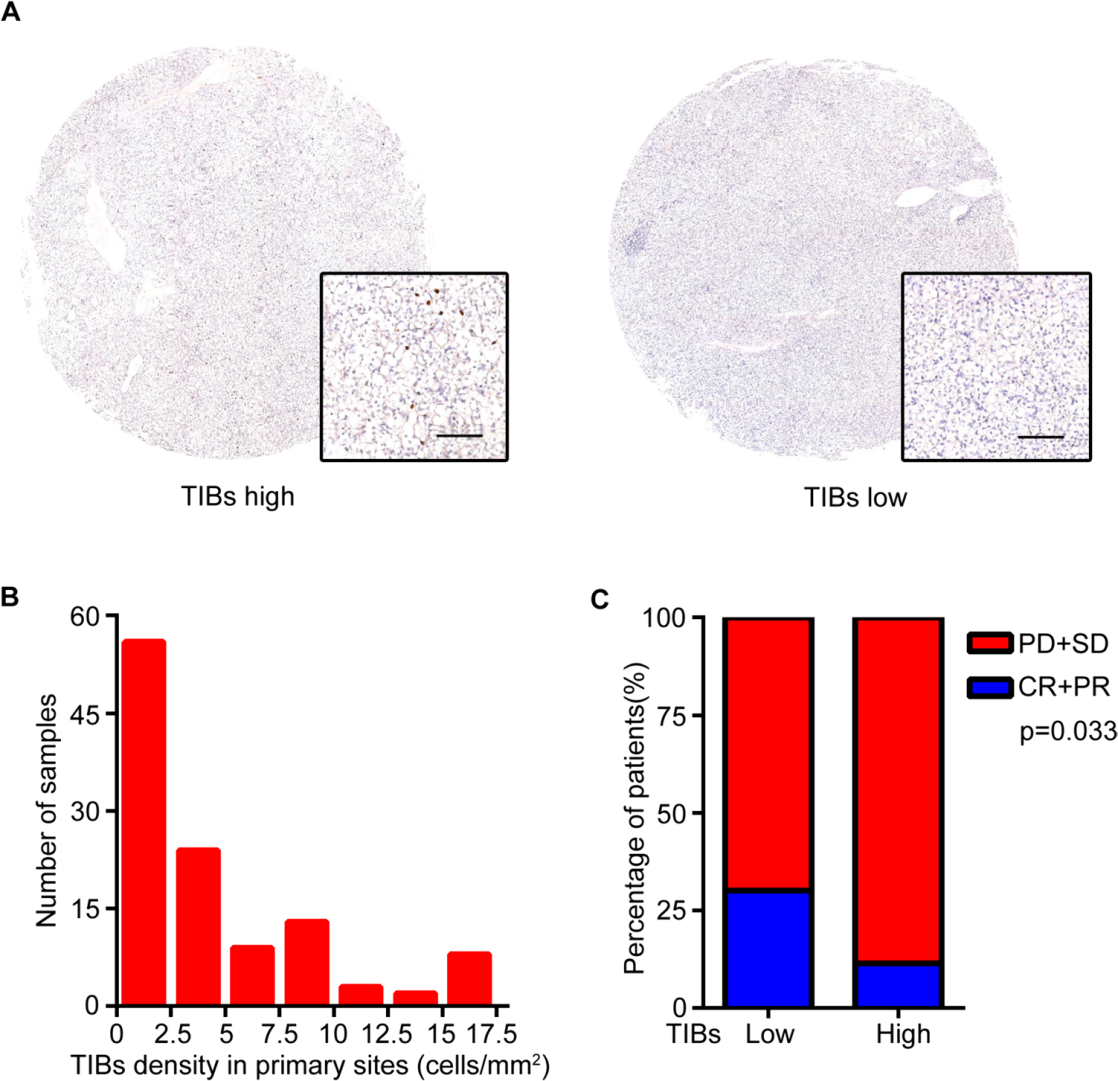
TIBs present in tumor samples and predict therapeutic response in mccRCC patients. **A** Representative images of CD19 staining in tumor samples. Scale bar is 50 μm. **B** Distribution of TIBs density in tumor samples of the study cohort. **C** Treatment response in different TIBs groups. *CR* complete response; *PR* partial response; *SD* stable disease; *PD* progressive disease

TIBs expression level and localization were evaluated by IHC staining on primary RCC specimens by using anti-CD19 antibody. As shown, CD19 was mainly distributed on the membrane of TIBs (Fig. 2A). The density of TIBs varied from 0/mm^2^ to 46/mm^2^ (Fig. 2B). TIBs infiltrating status had no correlation with gender, age, TNM stage, ISUP grade or metastatic organ number. Patients with high TIBs showed lower response to the combination therapy (p = 0.033) (Fig. 2C).

### TIBs portended shorter OS and PFS in combination treated ccRCC patients

**Fig. 3:**
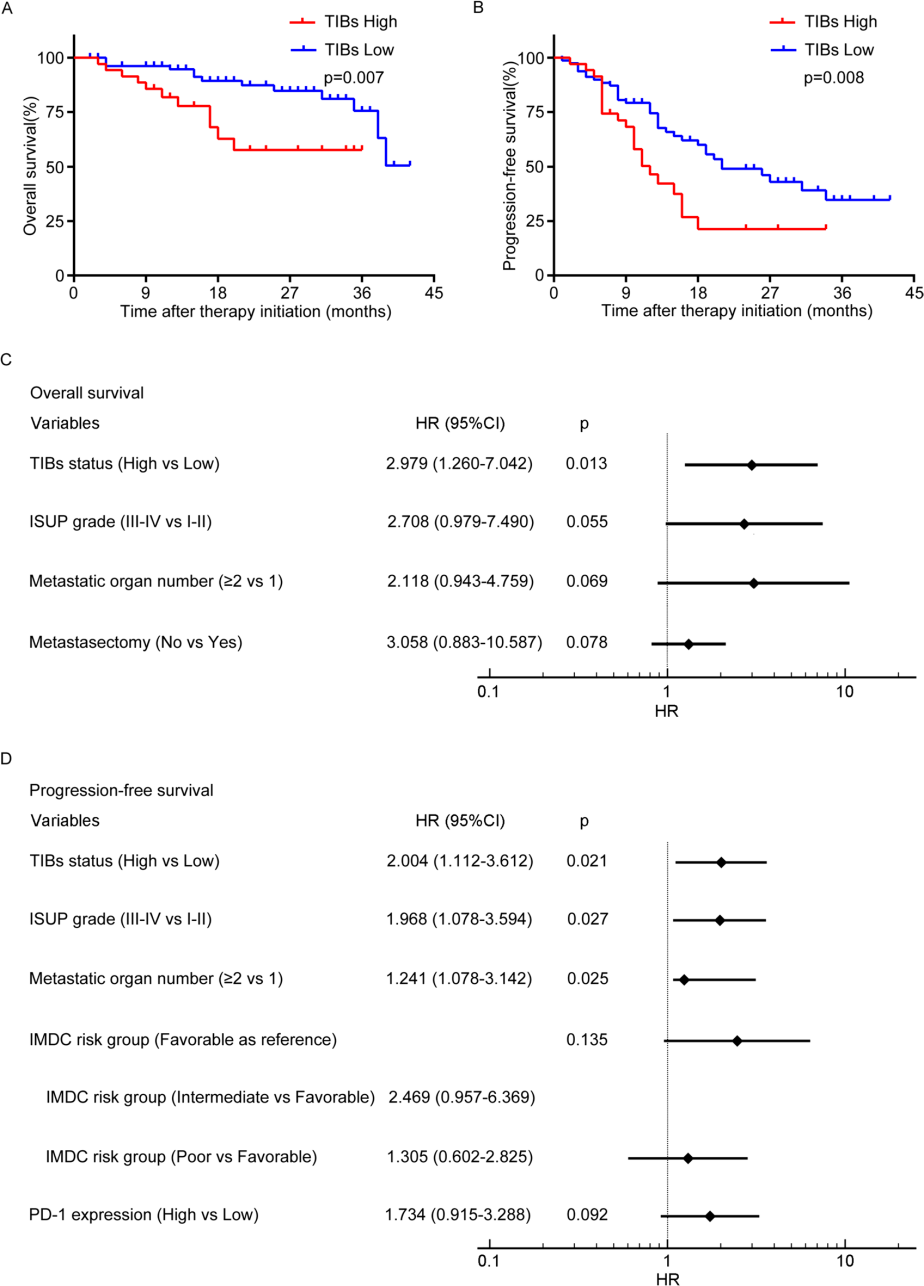
TIBs status predicts survival in mccRCC patients. **A**, **B** Kaplan–Meier survival analysis of OS (**A**) and PFS (**B**) according to TIBs grouping. **C**, **D** Multivariate analysis identified independent prognostic factors of OS (**C**) and PFS (**D**). *HR* hazard ratio; *CI* confidence interval

In the cohort, patients with high TIBs were linked with shorter OS by Kaplan–Meier analysis (p = 0.007) (Fig. 3A). Univariate Cox regression model revealed that ISUP grade (p = 0.007), TIBs (p = 0.01), metastatic organ number (p = 0.04) and received metastasectomy (p = 0.035) were significant prognostic factors for OS. Multivariate-Cox analysis confirmed TIBs was an independent predictor for poorer OS of combination treated ccRCC patients (p = 0.013) (Fig. 3C). The univariate- and multivariate-Cox analyses of TIBs and other clinical characteristics with OS were listed in Table S3.

At the final followup, patients with high TIBs demonstrated shorter PFS (p = 0.008) (Fig. 3B). ISUP grade (p = 0.001), TIBs (p = 0.011), IMDC risk stratification (p = 0.026) and metastatic organ number (p = 0.043) had significant relevance to PFS in univariate-Cox regression model. Besides, PD-1 expression showed moderate effects on PFS (p = 0.064) in univariate-Cox analysis, which also needs to be considered. Via multivariate-Cox analysis, we confirmed TIBs was still a significant predictor of PFS in ccRCC patients treated with combination therapy (p = 0.021) (Fig. 3D). The univariate- and multivariate-Cox analyses of TIBs and other clinical characteristics with OS and PFS were listed in Table S3.

Kaplan–Meier analysis showed no significant difference between the Tislelizumab-treated and Pembrolizumab-treated groups in OS (p = 0.944) (Fig.S1A) or PFS (p = 0.310) (Fig.S1B). The objective response rate (ORR) also showed no significant difference between the two groups (p = 0.898).

### TIBs infiltration associated with immunosuppressive microenvironment

**Fig. 4:**
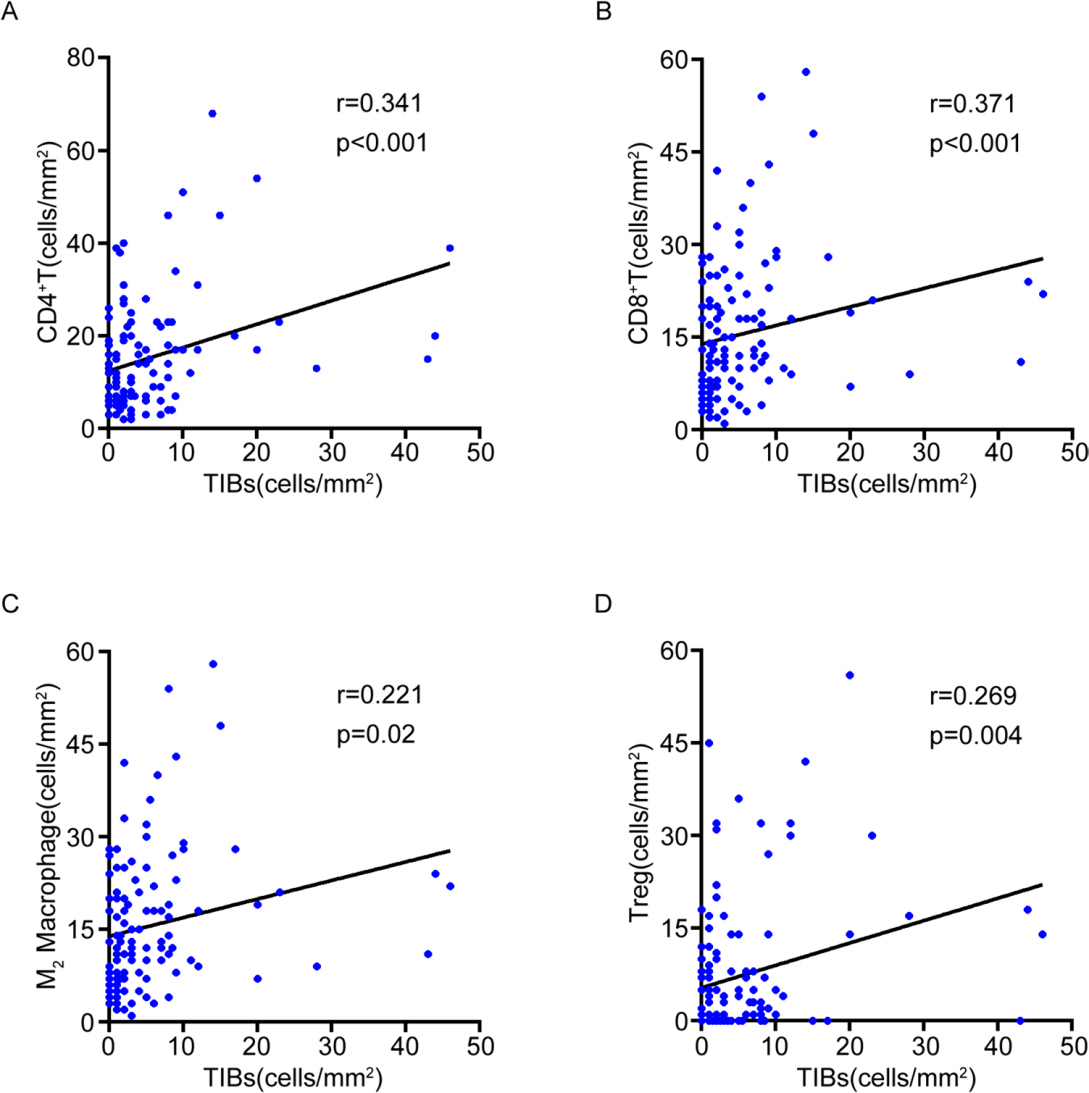
Correlation between TIBs and immune cells. **A**–**D** Pearson’s correlation between TIBs and CD4^+^T (A), CD8.^+^T (**B**), M2 macrophages (**C**) and Treg cells (**D**)

To investigate the potential mechanism of TIBs suppressive effect on combination therapy, we investigate immunocyte profiles in the cohort. IHC staining on microarray showed high TIBs associated with more infiltration of CD4^+^T (p < 0.001) (Fig. 4A), CD8^+^T (p < 0.001) (Fig. 4B), M2 macrophages (p = 0.020) (Fig. 4C) and regulatory T cells (Tregs) (p = 0.004) (Fig. 4D).

**Fig. 5:**
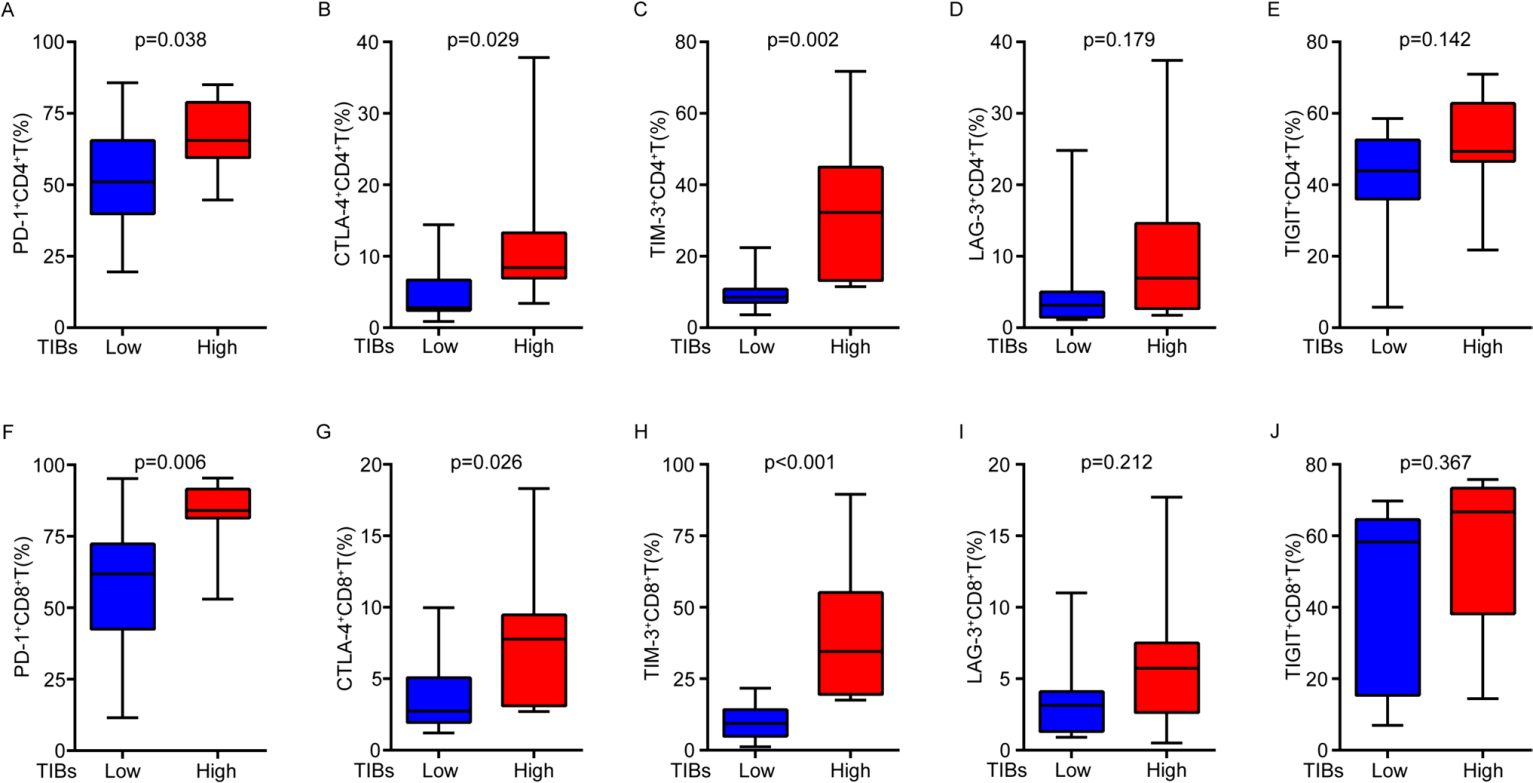
Comparison of immune checkpoints expression between TIBs high and low groups. **A**–**E** Comparison of PD-1 (**A**), TIM-3 (**B**), CTLA-4 (**C**), LAG3 (**D**) and TIGIT (**E**) positive rate in CD4^+^T cells between TIBs high and low groups. **F**–**J** Comparison of PD-1 (**F**), TIM-3 (**G**), CTLA-4 (**H**), LAG3 (**I**) and TIGIT (*J*) positive rate in CD8^+^T cells between TIBs high and low groups

In consideration of M2 macrophages and Treg cells exhaust CD8^+^T cells in tumor immune microenvironment (TIME), we explored the correlation between several immune checkpoints expression (PD-1, TIM-3, CTLA-4, LAG3 and TIGIT) and TIBs in the TCGA database. Results showed TIBs positive corrected with all these immune checkpoint molecules (Fig.S2). Then, we collected 22 fresh ccRCC samples and detected these immune checkpoints in CD4^+^T and CD8^+^T cells by FCM. Results showed that in TIBs high patients, PD-1, CTLA-4 and TIM-3 positive rate were significantly increased both in CD4^+^T (p = 0.038, 0.029 and 0.002 respectively, Fig. 5A–C) and CD8^+^T cells (p = 0.006, 0.026 and < 0.001 respectively, Fig. 5F–H). However, LAG3 and TIGIT expression level showed no significant difference in either CD4^+^T (p = 0.179 and 0.142 respectively, Fig. 5D–E) or CD8^+^T cells (p = 0.212 and 0.367 respectively, Fig. 5I, J).

## Discussion

ICIs plus TKIs has become first-line therapy for mRCC patients. Though, there is a lot of researches explored predictors in TKI or ICI monotherapy, little study has addressed predictors to guide selection for the combination therapy (Havel et al. 2019).

Emerging evidence shows TIME acts as a dominant force on treatment resistance, which can mediate immune evasion accounting for the interaction of immune cells and tumor cells (Barker et al. 2015; Correia and Bissell. 2012). Relieving TIME immunosuppressive effect is conducive to recover and reconstruct anti-tumor immunity, thereby enhancing the comprehensive therapeutic efficacy (Pitt et al. 2016). Hence, TIME has become an ideal target for cancer therapy (Belli et al. 2018; Roma-Rodrigues et al. 2019). The biological and clinical relevance of TIBs in RCC is still conflicting. Presence of CD19^+^B lymphocytes predicted better therapeutic response to sunitinib and longer survival (Lin et al. 2018). CD19^+^B cells also positive correlated with CD8^+^T cells in RCC. However, in another study, high infiltration of CD20^+^B cells indicated poor survival in RCC patients (Sjoberg et al. 2018). There was no study had yet explored the significance of TIBs in the ICIs plus TKIs combination therapy in RCC. This study showed high TIBs infiltration portended shorter OS (p = 0.007) (Fig. 3A) and PFS (p = 0.008) (Fig. 3B) in anti-PD-1 antibody plus Axitinib combination treatment. Multivariate cox regression analysis identified TIBs was an independent prognostic factor of OS (p = 0.013) (Fig. 3C) and PFS (p = 0.021) (Fig. 3D). Patients with high TIBs showed lower response to the combination therapy (p = 0.033) (Fig. 2C).

ccRCC belongs to immune “hot” tumors, which marked by large number of immune cells infiltrated in the TIME (Xu et al. 2023). In this study, high TIBs infiltration correlated with more CD4^+^T (p < 0.001) (Fig. 4A) and CD8^+^T cells (p < 0.001) (Fig. 4B) in RCC tumor sites. Unlike most tumors, high infiltration of CD8^+^T cells often predicts poor outcomes in RCC patients (Remark et al. 2013). It suggests that CD8^+^T cells infiltrated in RCC microenvironment are mostly functionally exhausted. Moreover, two immune suppressor cells, M2 macrophages (p = 0.020) (Fig. 4C) and Treg cells (p = 0.004) (Fig. 4D) were also increased in high TIBs patients.

Previous studies showed B cells could induce M2 macrophage polarization, as well as suppress CD8^+^T cells and M1 macrophages, which contributed the promotion of cancer cell proliferation (Liu et al. 2015; Roghanian et al. 2016). B cell-derived GABA promotes monocyte differentiation into anti-inflammatory macrophages that secrete interleukin-10 and inhibit CD8^+^T cell cytotoxic function (Zhang et al. 2021). Tumor associated macrophages can also upregulate the expression of inhibitory receptors PD-1 and CTLA-4 in T cells (Yin et al. 2019). Depletion of B cells prevented generation of M2 macrophage and increased the activity of antitumor T cell response (Affara et al. 2014; Liu et al. 2015).

High intratumoral B cells were also associated with increased recruitment and proliferation of Treg cells (Zhang et al. 2013). Bregs induce CD4^+^T cells to Tregs in IL-10 dependent and independent mechanisms (Zhang et al. 2013; X. Zhou et al. 2016a, b). IgD(low/-) B cells promote Treg homeostatic expansion via glucocorticoid-induced tumor necrosis factor receptor ligand (Ray et al. 2019). Tregs can also facilitate inhibitory receptor expression, including PD-1, on intratumoral T cells via enrichment of IL-35 expression (Sawant et al. 2019). Elimination of Tregs couled effectively restore IFN-γ production in the CD8^+^T cells and enhance the antitumor response to anti-VEGF therapy (Long et al. 2020).

In consideration of M_2_ macrophages and Treg cells exhaust CD8^+^T cells in TIME, we explored the correlation between several immune checkpoints expression (PD-1, TIM-3, CTLA-4, LAG3 and TIGIT) and TIBs in the TCGA database. Result showed TIBs positive corrected with all these immune checkpoint molecules (Supplemental Fig. 1). FCM verified that in TIBs high patients, PD-1, CTLA-4 and TIM-3 expression elevated significantly both in CD4^+^T (p = 0.038, 0.029 and 0.002 respectively, Fig. 5A–C) and CD8^+^T cells (p = 0.006, 0.026 and < 0.001 respectively, Fig. 5F–H). It suggested multiple immune checkpoints related exhaustion happened in these CD4^+^T and CD8^+^T cells. Only inhibiting PD-1 may inadequate to reverse these T cells exhaustion effectively. This explains low ORR of these patients to 天the combination treatment.

## Conclusions

In summary, our study revealed TIBs infiltration predicted adverse outcomes in mccRCC patients treated with anti-PD-1 antibody plus Axitinib. As a corollary, TIBs positively associated with M_2_ macrophages and Treg, leading to subsequent T cells exhaustion. Due to single-center and retrospective design, prospective or external validations is needed in order to strengthen evidence chain. Besides, the related mechanism have not been elaborated in this article and should be expound in-depth in future study. Nevertheless, our research is still helpful to understand the impact of TIBs in mccRCC patients treated with anti-PD-1 antibody plus Axitinib.

### Supplementary Information

Below is the link to the electronic supplementary material.

Supplementary file1 (DOCX 16 KB)

Supplementary file2 (DOCX 16 KB)

Supplementary file3 (DOCX 14 KB)

Supplementary file4 Fig.S2 Pearson’s correlation between TIBs and immune checkpoints expression from TCGA data. (A) Pearson’s correlation between TIBs and PD-1 expression. (B) Pearson’s correlation between TIBs and TIM-3 expression. (C) Pearson’s correlation between TIBs and CTLA-4 expression. (D) Pearson’s correlation between TIBs and LAG3 expression. (E) Pearson’s correlation between TIBs and TIGIT expression (TIF 204 KB)

Supplementary file5 Fig.S1 The Kaplan-Meier analysis of OS and PFS between Tislelizumab-treated and Pembrolizumab-treated patients. (A,B) Kaplan–Meier survival analysis of OS (A) and PFS (B) between Tislelizumab-treated and Pembrolizumab-treated patients (TIF 406 KB)

## Data Availability

The raw data used to support the findings of this study are available from the corresponding author upon reasonable request.
