# Supplementary material for: Tumor infiltrating B lymphocytes (TIBs) associate with poor clinical outcomes, unfavorable therapeutic benefit and immunosuppressive context in metastatic clear cell renal cell carcinoma (mccRCC) patients treated with anti-PD-1 antibody plus Axitinib
Source: J Cancer Res Clin Oncol. 2024 May 19;150(5):262. doi: 10.1007/s00432-024-05803-5 (PMC11102881; doi:10.1007/s00432-024-05803-5)
Supplement: Supplementary file 1 — Supplementary file1 (DOCX 16 KB) [file 432_2024_5803_MOESM1_ESM.docx]

Table S3 Univariate and multivariate analyses of TIBs and other clinical characteristics with OS and PFS

|  | OS (115 pts) | | | | | |  | PFS (115 pts) | | | | |
| --- | --- | --- | --- | --- | --- | --- | --- | --- | --- | --- | --- | --- |
| Characteristics | Univariate | |  | | Multivariate | |  | Univariate | |  | Multivariate | |
|  | HR (95% CI) | p | | HR (95% CI) | | p |  | HR (95% CI) | p | | HR (95% CI) | p |
| TIBs status |  | 0.010 | |  | | 0.013 |  |  | 0.011 | |  | 0.021 |
| High vs Low | 3.013 (1.298-6.994) |  | | 2.979 (1.260-7.042) | |  |  | 2.02 (1.179-3.481) |  | | 2.004 (1.112-3.612) |  |
| ISUP grade |  | 0.007 | |  | | 0.055 |  |  | 0.001 | |  | 0.027 |
| III-IV vs I-II | 3.926 (1.463-10.537) |  | | 2.708 (0.979-7.490) | |  |  | 2.470 (1.444-4.227) |  | | 1.968 (1.078-3.594) |  |
| Metastatic organ number |  | 0.040 | |  | | 0.069 |  |  | 0.043 | |  | 0.025 |
| ≥2 vs 1 | 2.328 (1.041-5.204) |  | | 2.118 (0.943-4.759) | |  |  | 1.713 (1.016-2.889) |  | | 1.241 (1.078-3.142) |  |
| Metastasectomy |  | 0.035 | |  | | 0.078 |  |  |  | |  |  |
| No vs Yes | 3.678 (1.096-12.341) |  | | 3.058 (0.883-10.587) | |  |  |  |  | |  |  |
| IMDC risk group |  |  | |  | |  |  |  | 0.026 | |  | 0.135 |
| Favorable |  |  | |  | |  |  | Reference |  | | Reference |  |
| Intermediate |  |  | |  | |  |  | 3.012 (1.209-7.519) |  | | 2.469 (0.957-6.369) |  |
| Poor |  |  | |  | |  |  | 1.309 (0.615-2.825) |  | | 1.305 (0.602-2.825) |  |
| PD-1 expression |  |  | |  | |  |  |  | 0.064 | |  | 0.092 |
| High vs Low |  |  | |  | |  |  | 1.777 (0.967-3.266) |  | | 1.734 (0.915-3.288) |  |

HR: hazard ratio. p<0.05 was regarded as statistically significant.
