# Supplementary material for: Tumor infiltrating B lymphocytes (TIBs) associate with poor clinical outcomes, unfavorable therapeutic benefit and immunosuppressive context in metastatic clear cell renal cell carcinoma (mccRCC) patients treated with anti-PD-1 antibody plus Axitinib
Source: J Cancer Res Clin Oncol. 2024 May 19;150(5):262. doi: 10.1007/s00432-024-05803-5 (PMC11102881; doi:10.1007/s00432-024-05803-5)
Supplement: Supplementary file 2 — Supplementary file2 (DOCX 16 KB) [file 432_2024_5803_MOESM2_ESM.docx]

Table S1 Immunohistochemistry (IHC) antibodies

| Identified cells/molecules | Antibody | Application | Manufacturer | Catalog No. | Diluted |
| --- | --- | --- | --- | --- | --- |
| B cells | Anti-CD19 antibody | IHC | Abcam | ab31947 | 1:200 |
| CD8^+^T cells | Anti-CD8α antibody | IHC | Abcam | ab199016 | 1:400 |
| CD4^+^T cells | Anti-CD4 antibody | IHC | Abcam | ab213215 | 1:50 |
| Tregs | Anti-FOXP3 antibody | IHC | Abcam | ab22510 | 1:1000 |
| Macrophages | Anti-CD68 antibody | IHC | Abcam | ab955 | 1:400 |
| M1 macrophages | Anti-CD86 antibody | IHC | Abcam | ab53004 | 1:500 |
| M2 macrophages | Anti-CD163 antibody | IHC | Abcam | ab111250 | 1:50 |
| PD-1^+^cells | Anti-PD1 antibody | IHC | Abcam | ab137132 | 1:1000 |
