# Supplementary material for: Tumor infiltrating B lymphocytes (TIBs) associate with poor clinical outcomes, unfavorable therapeutic benefit and immunosuppressive context in metastatic clear cell renal cell carcinoma (mccRCC) patients treated with anti-PD-1 antibody plus Axitinib
Source: J Cancer Res Clin Oncol. 2024 May 19;150(5):262. doi: 10.1007/s00432-024-05803-5 (PMC11102881; doi:10.1007/s00432-024-05803-5)
Supplement: Supplementary file 3 — Supplementary file3 (DOCX 14 KB) [file 432_2024_5803_MOESM3_ESM.docx]

Table S2 Flow cytometry (FCM) antibodies

| Marker | Antibody name | Clone | Manufacturer | Catalog No. |
| --- | --- | --- | --- | --- |
| CD8 | FITC anti-human CD8 Antibody | SK1 | BioLegend | 344704 |
| CD4 | APC/Cyanine7 anti-human CD4 Antibody | SK3 | BioLegend | 344616 |
| PD-1 | PE anti-human CD279 (PD-1) Antibody | NAT105 | BioLegend | 367404 |
| CTLA-4 | PerCP/Cyanine5.5 anti-human CD152 (CTLA-4) Antibody | BNI3 | BioLegend | 369608 |
| TIM-3 | PerCP/Cyanine5.5 anti-human CD366 (Tim-3) Antibody | F38-2E2 | BioLegend | 345016 |
| LAG-3 | PE anti-human CD223 (LAG-3) Antibody | 11C3C65 | BioLegend | 369306 |
| TIGIT | Brilliant Violet 421anti-human TIGIT(VSTM3) | A15153G | BioLegend | 372710 |
