# Supplementary figures and images for: Tumor infiltrating B lymphocytes (TIBs) associate with poor clinical outcomes, unfavorable therapeutic benefit and immunosuppressive context in metastatic clear cell renal cell carcinoma (mccRCC) patients treated with anti-PD-1 antibody plus Axitinib
Source: J Cancer Res Clin Oncol. 2024 May 19;150(5):262. doi: 10.1007/s00432-024-05803-5 (PMC11102881; doi:10.1007/s00432-024-05803-5)

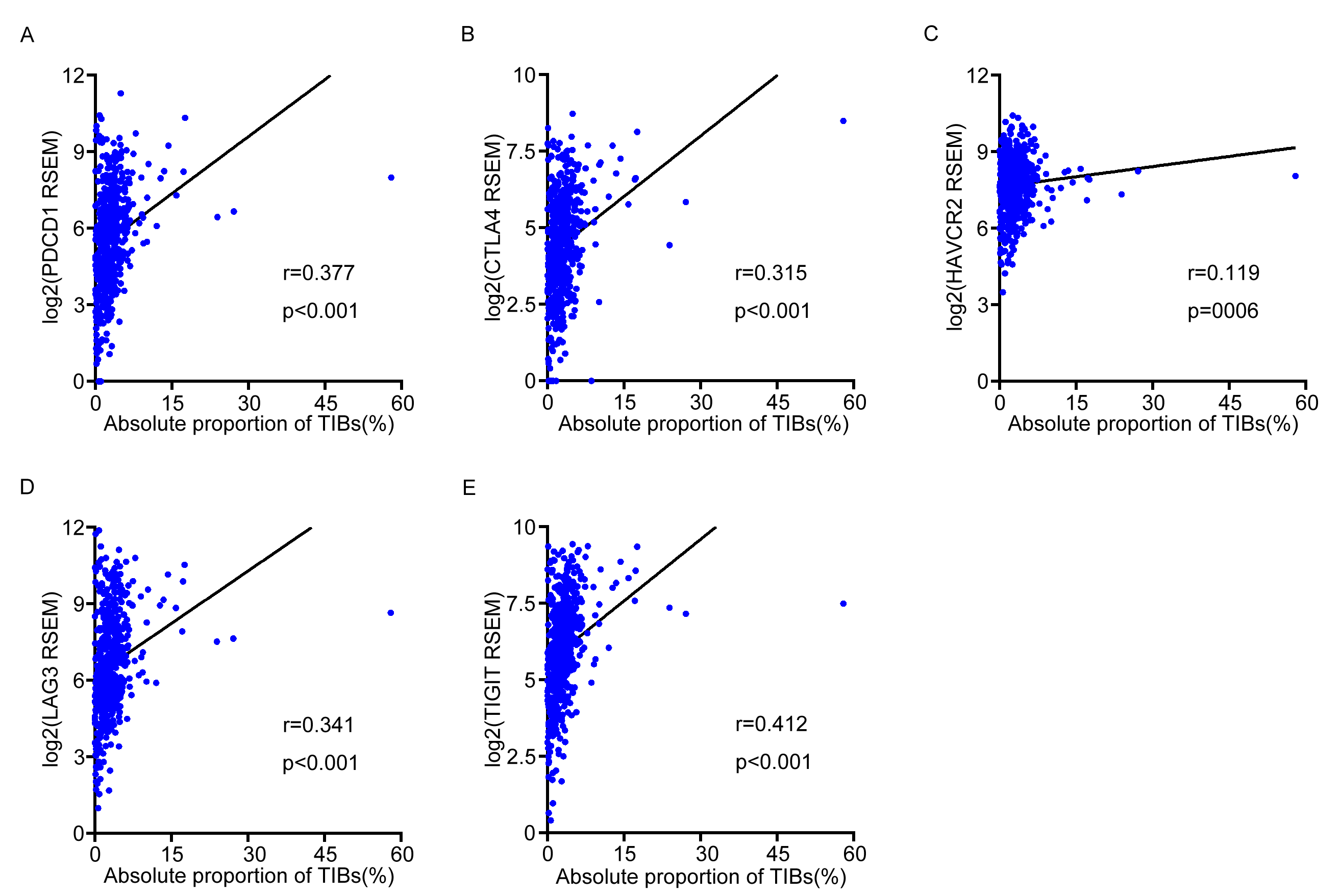

Supplement: Supplementary file 4 — Supplementary file4 Fig.S2 Pearson’s correlation between TIBs and immune checkpoints expression from TCGA data. (A) Pearson’s correlation between TIBs and PD-1 expression. (B) Pearson’s correlation between TIBs and TIM-3 expression. (C) Pearson’s correlation between TIBs and CTLA-4 expression. (D) Pearson’s correlation between TIBs and LAG3 expression. (E) Pearson’s correlation between TIBs and TIGIT expression (TIF 204 KB) [file 432_2024_5803_MOESM4_ESM.tif]

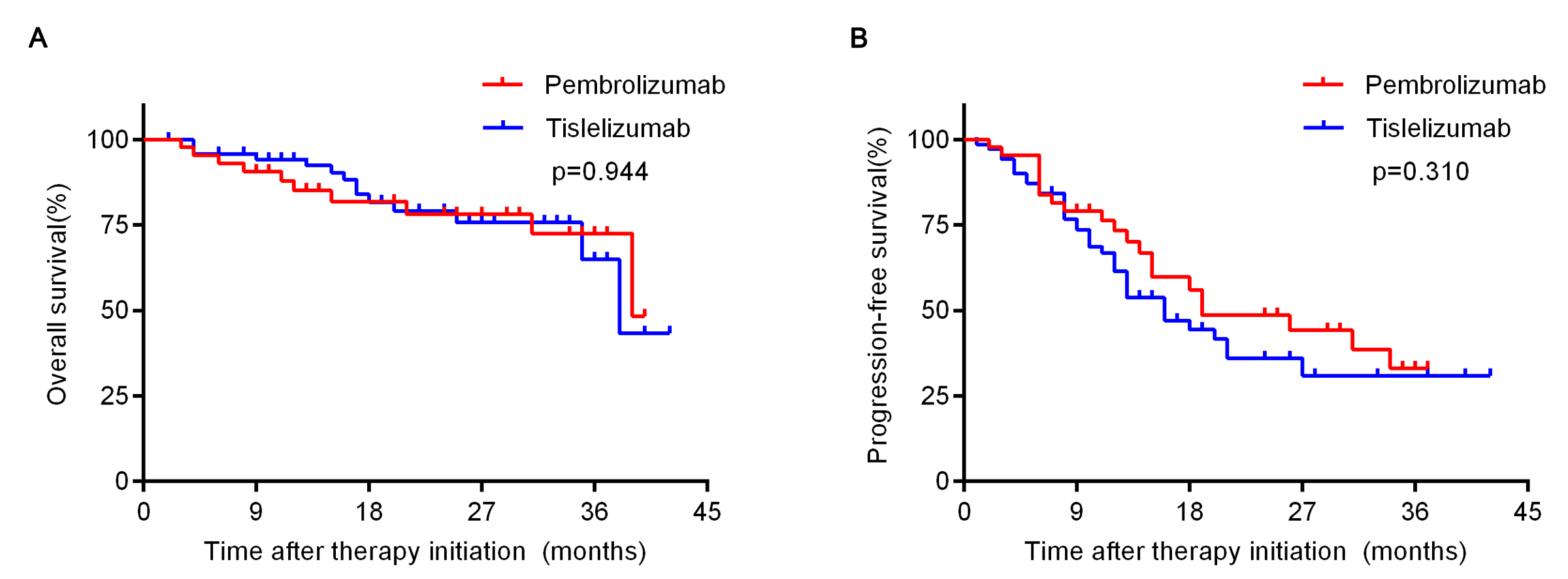

Supplement: Supplementary file 5 — Supplementary file5 Fig.S1 The Kaplan-Meier analysis of OS and PFS between Tislelizumab-treated and Pembrolizumab-treated patients. (A,B) Kaplan–Meier survival analysis of OS (A) and PFS (B) between Tislelizumab-treated and Pembrolizumab-treated patients (TIF 406 KB) [file 432_2024_5803_MOESM5_ESM.tif]
